# Beneficial effects of a nobiletin‐rich formulated supplement of Sikwasa (*C. depressa*) peel on cognitive function in elderly Japanese subjects; A multicenter, randomized, double‐blind, placebo‐controlled study

**DOI:** 10.1002/fsn3.2640

**Published:** 2021-10-26

**Authors:** Shizuo Yamada, Michiyo Shirai, Ken Ono, Toshiaki Teruya, Aki Yamano, Je Tae Woo

**Affiliations:** ^1^ Center for Pharma‐Food Research Graduate School of Pharmaceutical Sciences University of Shizuoka Shizuoka Japan; ^2^ Izu Health & Medical Center Shizuoka Japan; ^3^ Faculty of Education University of the Ryukyus Okinawa Japan; ^4^ Okinawa Research Center Co., Ltd Uruma Japan; ^5^ Department of Biological Chemistry College of Bioscience and Biotechnology Chubu University Kasugai Japan

**Keywords:** elderly subject, memory function, Nobiletin, WMS‐R

## Abstract

**Background:**

Nobiletin exerts beneficial effects on cognitive function in various animal models of Alzheimer's disease. The present study aimed to investigate the benefits and safety of a combination food of nobiletin‐rich extract from *C. depressa* peel for healthy elderly subjects.

**Methods:**

The nobiletin‐containing test food (Nobilex^®^) comprised high‐purity nobiletin powder combined with dried root powder of *K. parviflora* and dried lead powder of *P. japonicum* and was administered to elderly Japanese subjects once a day for 16 weeks. The Japanese version of the Wechsler Memory Scaled‐Revised (WMS‐R) was used as a primary evaluation item for the assessment of global memory. Data from a protocol‐matched population (Per Protocol Set: PPS) (*n* = 108) were analyzed.

**Results:**

The scores of “general memory” or “visual memory” in the indices of WMS‐R were significantly higher in the nobiletin‐containing test food group than in the placebo group. The difference in the total WMS‐R score was significantly higher in the test‐food group (9.0 ± 7.20) than in the placebo group (5.9 ± 7.70). An age‐stratified analysis of the WMS‐R test showed similar changes in subjects aged ≦74 years to those in the overall subject population. In the stratified analysis involving subjects with an MMSE‐J score of between 24 and 28, the “figural memory” subscale score in WMS‐R was significantly higher in the test food group than in the placebo group.

**Conclusion:**

The present results indicate that the nobiletin‐containing test food is beneficial for improving memory dysfunction in healthy elderly subjects.

## Graphical abstract

Nobiletin exerts beneficial effects on cognitive function in various animal models of Alzheimer's disease. The present results indicate that the nobiletin‐containing test food is beneficial for improving memory dysfunction in healthy elderly subjects.

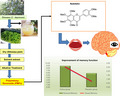

## 1 INTRODUCTION

The number of patients with dementia worldwide was 46.8 million in 2015 and is estimated to increase to 131.5 million by 2050 (Prince et al., 2015). In Japan, the incidence of cognitive/memory disorders, represented by dementia, has increased with the expanding elderly population. Alzheimer`s disease (AD), an age‐related neurodegenerative disorder, is the most common type of dementia. The pathological hallmarks of AD are accumulation of amyloid‐β (Aβ) plaques, neurofibrillary tangles, and neuronal loss (Jack et al., 2013). It is characterized by impaired cholinergic function in the brain, and donepezil has been used in the treatment of AD as a cholinergic enhancer to inhibit acetylcholinesterase (AChE), resulting in increased acetylcholine levels in synaptic clefts. However, since current medical treatments do not significantly attenuate the progression of this disease, more efficient therapeutic and preventive interventions are imperative for sustaining a healthy elderly population.

The development of brain function‐improving foods that may be safely consumed for prevention needs to be accelerated. Citrus fruits contain flavonoids that exhibit various physiological activities (Nogata et al., 2006). They have been utilized as fruits, foods, and supplements that may exert favorable effects on health. Among these flavonoids, the various physiological functions of polymethoxylated flavonoids (PMFs) have recently been investigated. Nobiletin, 3′,4′,5,6,7,8‐hexamethoxyflavone, is a representative PMF compound, that is abundant in the peels of citrus fruits, such as Sikwasa (*Citrus depressa*) (Nogata et al., 2006). We developed a method to manufacture material with high‐purity nobiletin and tangeretin from citrus peel, *C*. *depressa,* whose process includes dehydrating the peel, extracting the dried peel with ethanol, and treating extracts with alkaline (Woo, 2017). This material contains 65% nobiletin and 25% tangeretin, as the main components, with sinensetin as a minor component. Nobiletin has been reported to exert multiple biological effects, such as anti‐inflammatory, anti‐tumor, and neuroprotective activities (Choi et al., 2011; Lee et al., 2010; Miyamoto et al., 2008; Mulvihill et al., 2011); Roza et al., 2007; Tanaka et al., 2004; Yasunaga et al., 2016). The beneficial effects of nobiletin against neurodegenerative disorders, including AD, have recently been extensively reviewed (Braidy et al., 2017; Nakajima & Ohizumi, 2019). Furthermore, its beneficial effects on cognitive function in various animal models of AD have been characterized (Nakajima & Ohizumi, 2019; Onozuka et al., 2008). A human study on combination therapy with a dried nobiletin‐rich *C*. *reticulata* peel extract containing nobiletin and donepezil demonstrated significant improvements in the combination group relative to the donepezil‐treated group (Seki et al., 2013). A cohort study on elderly Japanese subjects by Zhang et al. (2017) revealed that the frequent consumption of citrus was associated with a reduced risk of the onset of dementia, even after adjustments for possible confounding factors.

An Okinawan material component, *Kaempferia parviflora* extract containing PMFs, was shown to reduce the cellular growth insufficiency at nerve regeneration sites in the hippocampus and thereby attenuate cognitive dysfunction (Plaingam et al., 2017). Furthermore, *Peucedam japonicum* extract, which has traditionally been used in Okinawa based on the assumption that the consumption of a stump each day may prolong the life by one day, exhibited antiobesity (Nugara et al., 2014) and anti‐inflammatory activities (Chun et al., 2018). Therefore, the present study aimed to investigate the benefits and safety of a combination food of nobiletin‐rich extract from *C. depressa* peel with extracts of *K. parviflora* and *P. japonicum*, in healthy elderly subjects with MMSE scores of between 24 and 30, and memory lapses. A randomized, double‐blind, placebo‐controlled study was conducted to establish whether this product promotes cognitive improvements in humans.

## 2 METHODS AND MATERIALS

### 2.1 Test food

Sikwasa (*C. depressa*) is a citrus fruit that grows naturally in the Northern area of Okinawa, with an annual harvest of approximately 4,000 t. A large portion is processed into juice, leaving behind tons of citrus peels as industrial waste. The Okinawa Research Center Organization established a method to manufacture high‐purity nobiletin powder (PMF90: nobiletin, 65%; tangeretin, 25%) from the residual by‐product, through isolation using ethanol extraction/concentration and an alkaline treatment (Woo, 2017). The test food enriched with high‐purity nobiletin powder, combined with extracts of *K*. *parviflora* and *P. japonicum,* is commercially available as a supplement (Nobilex^®^) to improve the quality of life (QOL) of the elderly. One capsule contains the dried powder extract (containing 10.0 mg nobiletin and 5.8 mg tangeretin) of *C. depressa* peel, the dried root powder (126.7 mg) of *K*. *parviflora* and the dried leaf powder of *P. japonicum* (33.3 mg). These plant extracts were not included in the placebo capsules. Three capsules of the nobiletin‐containing test food or placebo food were administered to each subject once a day for 16 weeks.

### 2.2 Study design

The study was conducted as a randomized, double‐blind, placebo‐controlled trial. Its protocol was approved as a specific clinical study by the Clinical Research Examination Ethical Committee of Hamamatsu Medical University on October 4, 2019, and registered to the Japan Registry of Clinical Trials (jRCT) (jRCTs041190073) on October 7, 2019. The present study was conducted in accordance with the Helsinki Declaration and “Clinical Research methods and regulations for Clinical Research methods”, considering subjects’ human rights and safety/welfare. Study participants were recruited by medical institutions and community organizations. The study contents were sufficiently explained to individuals wishing to participate in this study, and written informed consent was obtained. The study period was between October 7, 2019, and March 31, 2020. There were no changes in the protocol.

### 2.3 Subjects

The number of subjects was 122, consisting of healthy subjects who consulted the Seishukai Takido Medical Clinic, Shujikai Hagiwara Medical Clinic, or Izu Health & Medical Center as well as elderly residents of the area who were able to participate/cooperate. Primary selection criteria included healthy males and females, aged ≥65 years, with memory lapses and an MMSE‐J score of ≥24. After obtaining written informed consent on recruitment, subjects who met the selection criteria, but not the exclusion criteria, were selected.

### 2.4 Selection criteria

- Written informed consent was obtained from the individual before participating in this study.
- Individuals aged ≥65 years, with memory lapses.
- Individuals who were able to consume the test food during the study period.
- Individuals who were able to write a diary during the consumption period.
- Individuals with an MMSE‐J (Folstein et al., 1975) score of ≥24.

### 2.5 Exclusion criteria

- Patients with dementia.
- Individuals with a history of stroke, subarachnoid hemorrhage, cerebral infarction, cerebral hemorrhage, or brain contusion/head trauma requiring treatment/admission/surgery.
- Individuals with a history of epilepsy or diabetes or those being treated at outpatient clinics due to these diseases.
- Individuals with potential allergies to components of the test food.
- Individuals receiving drugs that may influence cognitive function (antipsychotic drugs, anxiolytics, antidepressants, anti‐Parkinson drugs, antimanic drugs, antiepileptic drugs, and drugs for dementia).
- Individuals participating or planning to participate in other clinical studies at the start of this study.
- Individuals considered to be ineligible by the chief investigator.

### 2.6 Randomization and blinding

The subjects of this study comprised 122 healthy elderly persons enrolled, and they were randomly assigned to a placebo or nobiletin‐containing test food group using the permuted block method. The assignment table was sealed, stored until the completion of this study, and opened after its completion. We confirmed that there were no significant differences in age or sex between the two groups.

### 2.7 Evaluation method

Testing was conducted at the start of consumption and after 16 weeks. Compliance was confirmed by telephone in the midterm of the present study. When the subject withdrew from this study or when test‐food consumption was discontinued, the study was completed. Furthermore, complications or adverse events were recorded based on reports from subjects. In subjects who had taken drugs for other diseases before the start of the present study, neither the dose nor dosing method was changed during the study period.

A primary evaluation criteria, the Japanese version of the Wechsler Memory Scale‐Revised (WMS‐R) (Neuner et al., 2007; Wechsler, 1987) consisted of 4 indices: general memory involving verbal and visual memories, and attention/concentration, and 8 subscales (information/orientation, figural memory, logical memory I, visual paired associates I, verbal paired associates I, visual reproduction I, number counting, and visual memory span) comprising the 4 indices. This scale facilitated the global memory assessment. We used 6 subscales in the present study, excluding “visual reproduction I” and “visual memory span.” As accessory evaluation criteria (cognitive function tests), we adopted MMSE‐J, the Benton Visual Retention Test, Symbol Digit Modalities Test, Cancellation and Detection‐Visual Cancellation Task, Trail Making Test (TMT)‐Japanese, General Health Questionnaire (GHQ)‐12 (Japanese version), and our original questionnaire. Although MMSE‐J is used appropriately for the screening of cognitive impairment and dementia in primary care, WMS‐R is more suitable for the systematic examination of memory function.

General examination parameters included height, body weight, body mass index (BMI), blood pressure, and pulse rate. Hematological parameters included the leukocyte count, erythrocyte count, hemoglobin (Hb), hematocrit (Ht), mean corpuscular volume (MCV), mean corpuscular hemoglobin (MCH), mean corpuscular hemoglobin concentration (MCHC), platelet count, uric acid, urea nitrogen, aspartate aminotransferase (AST)(GOT), alanine aminotransferase (ALT)(GPT), γ‐glutamyl transpeptidase (GTP), alkaline phosphatase (ALP), lactic acid dehydrogenase (LDH), total bilirubin, total protein, albumin, creatinine (Cre), estimated glomerular filtration rate (e‐GFR), creatine phosphokinase (CPK), serum amylase, total cholesterol, high‐density lipoprotein (HDL)‐cholesterol, low‐density lipoprotein (LDL)‐cholesterol, neutral fat, blood glucose, HbA1c, Na, Cl, K, Mg, Ca, Fe, testosterone, and adiponectin (LA). In the urinalysis, qualitative protein/glucose/urobilinogen/bilirubin/occult blood reaction/ketone body tests, as well as specific gravity and pH tests were conducted. Blood, urine, and cognitive function tests were generally conducted twice at the start of food consumption and after 16 weeks. Safety assessment items included the number of patients with adverse events, adverse reactions, severe adverse events, or severe adverse reactions/their contents, and the number of patients in whom the oral administration of drugs for dementia was started/their contents. These items were evaluated based on the results of general and blood examinations.

### 2.8 Statistical analysis

To compare results between the test‐food and placebo groups, differences in scores after the completion of test food consumption from the baseline scores of the respective examination items were investigated using the unpaired *t*‐test. When comparing baseline values between the two groups, sex/age was compared using the chi‐squared test and the results of cognitive function tests using the unpaired *t*‐test. We used SPSS Statistics, ver. 25 software. In all tests, two‐sided tests were adopted. A *p*‐value of .05 was considered to indicate significant difference.

Since the WMS‐R test is recommended for individuals aged ≤75 years, a stratified analysis was performed by dividing subjects into two age groups consisting of those aged 65 to 74 years and those aged ≥75 years, based on the number of subjects. Furthermore, a stratified analysis was conducted by dividing subjects into two groups based on the baseline MMSE‐J score (between 24 and 28 and ≥29) for comparisons.

## 3 RESULTS

### 3.1 Study participants

**FIGURE 1:**
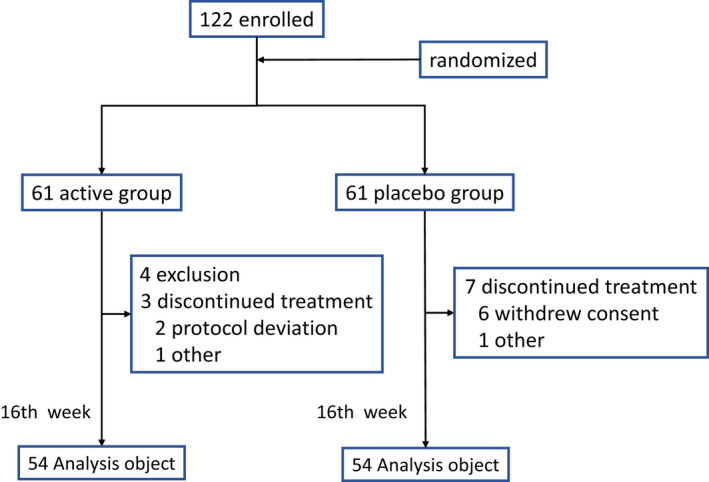
Flow diagram of subjects throughout the study

A flow chart of subjects is shown in Figure 1. The present study was conducted without changing its protocol. Subjects consisted of 38 males and 84 females (total: 122), and they were randomized as described in the Methods. Finally, we analyzed the data from a protocol‐matched population (Per Protocol Set: PPS) (*n* = 108), excluding 4 subjects meeting the exclusion criteria (“exclusion” in Figure 1), 6 subjects who withdrew from the study through discontinuation after the intervention (“withdrew consent” in Figure 1), 2 subjects who were considered to be ineligible by physicians after the intervention (“other” in Figure 1), and 2 subjects with missing data on the main evaluation items (“protocol deviation” in Figure 1). The safety analysis set (SAS) comprised 117 subjects, the full analysis set (FAS) comprised 115 subjects, and the PPS comprised 108 subjects.

Regarding sex, there were 39 females and 15 males in the nobiletin‐containing test food group, and 36 females and 18 males in the placebo group. The nobiletin‐containing test food group consisted of 18 subjects aged 65 to 69 years, 22 subjects aged 70 to 74 years, 9 subjects aged 75 to 79 years, and 5 subjects aged ≥80 years, while the placebo group comprised 17 subjects aged 65 to 69 years, 14 subjects aged 70 to 74 years, 15 subjects aged 75 to 79 years, and 8 subjects aged ≥80 years. The baseline characteristics of both groups are shown in Table 1. No significant differences were observed in any variable between these groups.

**TABLE 1:** Subject backgrounds

Variable	Active group (*n* = 54)		Placebo group (*n* = 54)		*p* value
	Mean	*SD*	Mean	*SD*	
Sex (male/female)	15/39		18/36		.53
Age (years)	73.3	5.3	72.2	5.1	.29
Height (cm)	155.2	8.0	154.7	7.9	.75
Weight (kg)	55.2	8.9	56.6	9.7	.44
BMI (kg/m2)	22.9	2.9	23.6	3.6	.25
Blood pressure (mmHg)					
Systolic	138.1	20.4	145.2	21.0	.08
Diastolic	76.3	11.9	78.8	12.2	.30
Pulse	74.6	12.1	73.1	13.1	.53
Cognitive function					
MMSE‐J	28.9	1.3	28.6	1.3	.26
WMS‐R (0 min)	68.1	13.7	66.8	13.4	.61
BVRT	5.7	1.6	5.6	1.5	.76
SDMT	42.7	7.4	41.2	8.2	.32
CD‐VCT time(sec)	101.5	23.1	108.1	22.3	.13
CD‐VCT response rate (%)	98.7	1.6	98.5	2.1	.62
TMT	51.2	20.0	52.2	15.9	.77
GHQ−12	37.2	4.3	37.3	3.5	.88
QOL survey	18.2	4.2	17.6	3.9	.45
Blood biochemistry					
Alb (albumin)(g/dl)	4.3	0.2	4.2	0.6	.24
AST(GOT) (U/L)	25.5	10.9	23.9	8.9	.40
ALT(GPT) (U/L)	21.6	18.4	20.9	11.3	.82
γ‐GTP (IU/L)	29.4	20.1	33.6	56.8	.61
Total cholesterol (mg/dl)	216.4	37.8	210.0	39.8	.40
Triglyceride (mg/dl)	145.4	88.2	150.0	89.8	.79
HDL cholesterol (mg/dl)	64.0	16.3	61.7	18.5	.49
LDL cholesterol (mg/dl)	121.2	32.1	119.7	32.9	.81
Glucose (mg/dl)	112.8	29.5	115.4	27.5	.64
HbA1c (NGSP) (%)	5.8	0.4	5.7	0.3	.40
eGFR (ml/min/1.73 m^2^)	68.3	13.7	69.3	13.1	.72
Blood urea nitrogen (mg/dl)	16.5	4.3	15.7	3.6	.29
Creatinine (mg/dl)	0.7	0.2	0.7	0.2	.82
Adiponectin (μg/ml)	13.7	7.4	13.1	7.3	.67

Note

Differences between the placebo and active groups were analyzed using the chi‐squared test for proportions and the unpaired *t*‐test for means.

Abbreviations: ALT, Alanine aminotransferase; AST, Aspartate aminotransferase; BMI, body mass index; BVRT, Benton Visual Retention Test; CD‐VCT, Cancellation and Detection (Visual Cancellation Task); eGFR, estimated glomerular filtration rate; GHQ12, General Health Questionnaire; HbA1c(NGSP), Hemoglobin A1c (National Glycohemoglobin Standardization Program); HDL, High‐density lipoprotein; LDL, Low‐density lipoprotein; min, minute; MMSE‐J, Mini Mental State Examination‐Japanese; SDMT, Symbol Digit Modalities Test; sec, second; TMT, Trail Making Test‐Japanese; WMS‐R, Wechsler Memory Scale‐Revised; γ‐GTP, Gamma‐Glutamyl Transpeptidase.

### 3.2 Cognitive function assessment

The results of the WMS‐R test involving the primary evaluation items are shown in Tables 2 and 3. Differences in the mean of each cognitive function test between the baseline and evaluation points were compared. As shown in Table 2, the difference in the total WMS‐R score was significantly higher in the test food group (9.0 ± 7.20) than in the placebo group (5.9 ± 7.70). The average actual values for the nobiletin‐containing test food group and placebo group were 68.1 and 66.8 before (baseline), respectively, and 77.1 and 72.7, respectively, after the repeated treatment for 16 weeks.

**TABLE 2:** Mean difference from the baseline for cognitive function tests

Variable	Active group (*n* = 54)			Placebo group (*n* = 54)			*p* value
	Mean	*SD*	(95%CI)	Mean	*SD*	(95%CI)	
MMSE‐J	0.35	1.29	(0.0;0.7)	0.46	1.45	(0.86;0.07)	.68
WMS‐R (0 min)	9.00	7.20	(7.01;10.92)	5.90	7.70	(3.83;8.02)	.04
BVRT	0.57	1.75	(0.1;1.05)	0.57	1.37	(0.2;0.95)	1.00
SDMT	1.41	4.16	(0.27;2.54)	0.82	4.31	(−0.35;2.0)	.48
CD‐VCT time (sec)	−7.61	17.40	(−12.36;−2.86)	−4.52	16.55	(−9.04;0.0)	.35
CD‐VCT response rate (%)	0.06	1.53	(−0.35;0.48)	0.46	1.86	(−0.05;0.97)	.23
TMT	−3.39	22.91	(−9.64;2.87)	−4.78	10.95	(−7.77;−1.79)	.69
GHQ−12	0.94	3.67	(−0.06;1.95)	0.39	3.45	(−0.55;1.33)	.42

Note

Differences between the placebo and active groups were analyzed using unpaired *t*‐test for means.

Abbreviations: BVRT, Benton Visual Retention Test; CD‐VCT, Cancellation and Detection (Visual Cancellation Task); GHQ12, General Health Questionnaire; Min, minute; MMSE‐J, Mini Mental State Examination‐Japanese; SDMT, Symbol Digit Modalities Test; Sec, second; TMT, Trail Making Test‐Japanese; WMS‐R, Wechsler Memory Scale‐Revised.

**TABLE 3:** Mean difference from the baseline for indices of WMS‐R

Variable	Active group (*n* = 54)			Placebo group (*n* = 54)			*p* value
	Mean	*SD*	(95%CI)	Mean	*SD*	(95%CI)	
General memory	8.91	6.82	(7.04;10.77)	5.87	6.99	(3.96;7.78)	.02
Visual memory	2.52	4.64	(1.25;3.78)	0.74	3.77	(−0.29;1.77)	.03
Verbal memory	6.39	6.08	(4.73;8.05)	5.13	5.94	(3.51;6.75)	.28
Attention/concentration	0.06	2.10	(−0.52;0.63)	0.06	2.75	(−0.7;0.81)	1.00

Note

Differences between the placebo and active groups were analyzed using unpaired *t*‐test for means.

The values of the indices are shown in Table 3. In the WMS‐R test, 5 indices can be calculated from the results of 8 subtests. The 5 indices consist of “general memory,” “visual memory,” “verbal memory,” “attention/concentration,” and “delayed recall.” In the present study, 4 index scores, excluding “delayed recall,” were obtained (Table 3). Furthermore, 6 subscale scores, excluding “visual reproduction” and “visual memory span”, were assessed. In comparisons of the results of the WMS‐R test between the test food and placebo groups, the “general memory” index score was significantly higher in the former than in the latter (Table 3), while no significant differences were noted in any subscale score (data not shown). A significant difference was observed in the “visual memory” score comprising “general memory” (Table 3).

The age‐stratified analysis of the WMS‐R test showed that changes observed in subjects aged ≤74 years were similar to those in the overall subject population. Scores of “general memory” or “visual memory” in the indices of WMS‐R were significantly higher in the nobiletin‐containing test food group than in the placebo group (Table 4). Furthermore, the “visual paired associates” subscale score in the subscales of WMS‐R was significantly higher in the former than in the latter (Table 4). In the stratified analysis involving subjects with an MMSE‐J score between 24 and 28, the “figural memory” subscale score in WMS‐R was significantly higher in the test food group than in the placebo group (Table 5). On the other hand, no significant differences were observed in any examination item in the subject population with an MMSE‐J score of ≥29 (data not shown).

**TABLE 4:** Mean difference from the baseline for cognitive function tests, indices, and subscales of WMS‐R in subjects younger than 74 years old (age≤74 years)

Variable	Active group (*n* = 40)			Placebo group (*n* = 31)			*p* value
	Mean	*SD*	(95%CI)	Mean	*SD*	(95%CI)	
**Cognitive tests**							
MMSE‐J	0.2	1.22	(−0.19;0.59)	0.65	1.14	(0.23;1.06)	.12
WMS‐R (0 min)	9.1	7.52	(6.69;11.51)	5.42	7.12	(2.81;8.03)	.04
BVRT	0.58	1.87	(−0.02;1.17)	0.32	1.45	(−0.21;0.85)	.54
SDMT	1.43	4.14	(0.10;2.76)	0.65	3.38	(−0.58;1.89)	.4
CD‐VCT time(sec)	−6.43	12.08	(−10.29;−2.56)	−3.42	10.72	(−7.35;0.51)	.28
CD‐VCT response rate (%)	0	1.45	(−0.47;0.46)	0.57	1.51	(0.02;1.13)	.11
TMT	−3.6	23.16	(−11.01;3.81)	−0.81	8.17	(−3.80;2.19)	.52
GHQ−12	1.05	3.35	(−0.02;2.12)	−0.42	2.99	(−1.51;0.68)	.06
<**Indices of WMS‐R>**							
General memory	8.95	7.15	(6.66;11.24)	5.39	6.81	(2.89;7.88)	.04
Visual memory	2.6	4.49	(1.16;4.04)	0.06	3.49	(−1.22;1.35)	.01
Verbal memory	6.35	5.89	(4.47;8.23)	5.32	5.92	(3.15;7.49)	.47
Attention/Concentration	0.15	2.18	(−0.55;0.85)	0.03	2.95	(−1.05;1.11)	.85
**<Subscales of WMS‐R>**							
Mental control	0.05	1.04	(−0.28;0.38)	−0.1	1.35	(−0.59;0.40)	.61
Figural memory	0.55	1.65	(0.02;1.08)	0.03	1.52	(−0.52;0.59)	.18
Logical memory	5.35	4.69	(3.85;6.85)	3.48	4.56	(1.81;5.16)	.1
Visual paired associates	2.05	4.28	(0.68;3.42)	0.03	3.5	(−1.25;1.32)	.04
Verbal paired associates	1	3.2	(−0.02;2.02)	1.84	3.08	(0.71;2.97)	.27
Digit SPAN	0.1	1.89	(−0.51;0.71)	0.13	2.33	(−0.73;0.99)	.95

Note

Differences between the placebo and active groups were analyzed using unpaired *t*‐test for means.

General memory: visual memory and verbal memory, visual memory: figural memory and visual paired associates, verbal memory: logical memory and verbal paired associates, attention/concentration: mental control and digit span.

Abbreviations: BVRT, Benton Visual Retention Test; CD‐VCT, Cancellation and Detection (Visual Cancellation Task); GHQ12, General Health Questionnaire; Min, minute; MMSE‐J, Mini Mental State Examination‐Japanese; SDMT, Symbol Digit Modalities Test; Sec, second; TMT, Trail Making Test‐Japanese; WMS‐R, Wechsler Memory Scale‐Revised.

**TABLE 5:** Mean difference from the baseline for subscales of WMS‐R, in subjects with a MMES‐J score of between 24 and 28

Variable	Active group (*n* = 17)			Placebo group (*n* = 21)			*p* value
	Mean	*SD*	(95%CI)	Mean	*SD*	(95%CI)	
Mental control	0.29	1.31	(−0.38;0.97)	−0.19	1.47	(−0.86;0.48)	.30
Figural memory	0.94	1.14	(0.35;1.53)	−0.10	1.64	(−0.84;0.65)	.03
Logical memory	6.24	5.17	(3.58;8.89)	4.52	3.68	(2.85;6.2)	.24
Visual paired Associates	1.94	4.68	(−0.47;4.35)	0.86	4.64	(−1.25;2.97)	.48
Verbal paired associates	1.53	3.52	(−0.28;3.34)	1.62	3.23	(0.15;3.09)	.94
Digit span	−0.35	1.46	(−0.35;−1.1)	0.24	2.55	(−0.92;1.4)	.38

Note

Differences between the placebo and active groups were analyzed using unpaired *t*‐test for means.

Regarding accessory evaluation items (MMSE‐J, Benton Visual Retention Test, Symbol Digit Modalities Test, Cancellation and Detection‐Visual Cancellation Task, TMT‐Japanese, GHQ‐12‐Japanese, and our original questionnaire), no significant differences were noted between the test food and placebo groups (Tables 2 and 4).

### 3.3 Safety assessment

No significant differences were observed in any parameter in general/blood/urine examinations before and after food consumption between the test food and placebo groups. There were also no adverse events.

## 4 DISCUSSION

Natural medicines have gained popularity in many Western countries in recent years due to their therapeutic effects and affordability for the general public (Rao et al., 2019). With the increasingly extensive application of natural medicines and their bioactive ingredients for disease treatment, clinical studies are imperative to ensure their efficacy and safety.

AD is characterized by impaired cholinergic function. Several anticholinesterase inhibitors, such as donepezil, are clinically used in the treatment of patients with AD. Memory dysfunction generally precedes other cognitive domain dysfunctions in the prodromal phase of AD (Petersen et al., 1999), which is also known as MCI. Memory deficits in neurological and psychiatric patients have been evaluated using neuropsychological tests, such as WMS‐R (Neuner et al., 2007; Wechsler, 1987), which is one of the more frequently used measures of memory and attention (Elwood, 1991; Kinno et al., 2017). Niwa et al. (2016) reported that the relationship between WMS‐R scores and a decreased regional cerebral blood flow (rCBF) may be important in cognitive assessments of elderly individuals because memory dysfunction precedes other cognitive dysfunctions with prodromal AD (Petersen et al., 1999). The present study assessed the effects of nobiletin‐containing test food on the functions of various memory domains in healthy elderly Japanese subjects using the primary outcome, the WMS‐R score. The results obtained revealed that in comparison with the placebo treatment, the efficacy of the repeated ingestion of nobiletin‐containing test food for improving memory function based on WMS‐R scores among healthy elderly subjects, was significantly greater, resulting in significant between‐treatment differences (Table 2). The consumption of the test food effectively improved the “visual memory” score (Table 3), suggesting the efficacy of visual care support for improving cognitive function.

In a subgroup analysis of these subjects, improvements in WMS‐R were more evident in subjects younger than 75 years than in those older than 75 years (Table 4). In the MMSE‐J score‐stratified analysis, no significant differences were observed in the population with an MMSE‐J score of ≥29, whereas a significant difference was noted in cognitive function in the population with an MMSE‐J score of between 24 and 28 (Table 5). Therefore, the nobiletin‐containing test food may exert beneficial effects in a a group with mild MCI and memory lapses. To the best of our knowledge, the present study is an unprecedented trial that addressed the efficacy of the repeated intake of nobiletin‐containing food on cognitive function in healthy elderly subjects assessed by WMS‐R. The results obtained appears to be supported by the efficacy of nobiletin against neurodegenerative disorders, such as AD (Braidy et al., 2017; Nakajima & Ohizumi, 2019). Notably, nobiletin exerted memory‐improving effects in a number of animal models of AD such as olfactory‐bulbectomized mice, Aβ‐infused rats, MK‐801‐treated mice, senescence‐accelerated mice, amyloid precursor protein Tg mice, and a rat model of cerebral ischemia Nakajima and Ohizumi (2019). Nobiletin has been shown to exert neuroprotective effects in several in vitro and in vivo studies, by attenuating cholinergic deficits, reducing the abnormal accumulation of neurotoxic amyloid‐beta peptides, reversing N‐methyl‐D‐aspartate (NMDA) receptor hypofunction, ameliorating ischemic injury, inhibiting the hyperphosphorylation of tau protein, suppressing β‐secretase, increasing neprilysin levels, and modulating several signaling cascades (Braidy et al., 2017; Nakajima & Ohizumi, 2019; Onozuka et al., 2008; Yasuda et al., 2014; Youn et al., 2017). Additionally, the significant distribution of nobiletin in the brain was detected by PET imaging of [^11^C]‐labeled synthetic nobiletin following an intravenous injection in rats (Asakawa et al., 2011) and by the direct measurement of its brain content in nobiletin‐administered mice (Saigusa et al., 2011), suggesting the blood‐brain permeability of nobiletin. The efficacy and safety of nobiletin‐rich *C*. *reticulata* peel extract for cognitive function were briefly reported in an intervention study on patients with AD by Seki et al. (2013); cognitive function in six patients with mild‐to‐moderate AD who were already administered donepezil was assessed with MMSE and the Japanese version of the Alzheimer's Disease Assessment Scale‐Cognitive Subscale (ADAS‐J cog). A one‐year intervention with nobiletin‐rich *C*. *reticulata* peel extract (*Chinpi* in Kampo medicine) was shown to significantly prevent the progression of cognitive impairments in these patients with no adverse events (Seki et al., 2013). In the present study, the nobiletin‐containing test food contains the extracts of *K*. *parviflora* and *P*. *japonicum*. The *K*. *parviflora* extract attenuated cell growth insufficiency at the site nerve of regeneration in the hippocampus (Plaingam et al., 2017). The *P*. *japonicum* extract may prolong life by one day due to its anti‐obesity (Nugara et al., 2014) and anti‐inflammatory activities (Chun et al., 2018). However, the ingredients exhibiting beneficial effect on the memory dysfunction have not been identified in the extracts of *K. parviflora* and *P. japonicum*.

## 5 CONCLUSIONS

In summary, we demonstrated for the first time that nobiletin‐containing test food may be beneficial for improving memory dysfunction in healthy elderly subjects.

## CONFLICT OF INTEREST

All authors declare that they have no conflicts of interest. All authors have approved the final draft of the manuscript.

## AUTHOR CONTRIBUTIONS

**Shizuo Yamada:** Conceptualization (equal); Data curation (equal); Funding acquisition (equal); Investigation (equal); Methodology (equal); Project administration (equal); Resources (equal); Supervision (equal); Validation (equal); Visualization (equal); Writing‐original draft (equal); Writing‐review & editing (equal). **Michiyo Shirai:** Data curation (equal); Formal analysis (equal); Methodology (equal); Project administration (equal); Writing‐original draft (equal). **Ken Ono:** Conceptualization (equal); Data curation (equal); Investigation (equal); Project administration (equal); Supervision (equal); Validation (equal); Visualization (equal). **Toshiaki Teruya:** Conceptualization (equal); Project administration (equal); Software (equal); Supervision (equal); Visualization (equal). **Aki Tamano:** Data curation (equal); Formal analysis (equal); Methodology (equal); Software (equal); Visualization (equal). **Woo Je‐Tae:** Conceptualization (equal); Funding acquisition (equal); Investigation (equal); Project administration (equal); Resources (equal); Software (equal); Supervision (equal); Validation (equal); Visualization (equal); Writing‐original draft (equal); Writing‐review & editing (equal).

## ETHICAL REVIEW

This study was approved by the Clinical Research Examination Ethical Committee of Hamamatsu Medical University on October 4, 2019. The study conforms to the Declaration of Helsinki, US, and/or European Medicines Agency Guidelines for human subjects.
